# An Ill Wind? Climate Change, Migration, and Health

**DOI:** 10.1289/ehp.1104375

**Published:** 2012-01-20

**Authors:** Celia McMichael, Jon Barnett, Anthony J. McMichael

**Affiliations:** 1 School of Social Sciences, La Trobe University, Melbourne, Australia; 2 Department of Resource Management and Geography, University of Melbourne, Melbourne, Australia; 3 National Centre for Epidemiology and Population Health, Australian National University, Canberra, Australia

**Keywords:** climate change, displacement, health, migration, resettlement

## Abstract

Background: Climate change is projected to cause substantial increases in population movement in coming decades. Previous research has considered the likely causal influences and magnitude of such movements and the risks to national and international security. There has been little research on the consequences of climate-related migration and the health of people who move.

Objectives: In this review, we explore the role that health impacts of climate change may play in population movements and then examine the health implications of three types of movements likely to be induced by climate change: forcible displacement by climate impacts, resettlement schemes, and migration as an adaptive response.

Methods: This risk assessment draws on research into the health of refugees, migrants, and people in resettlement schemes as analogs of the likely health consequences of climate-related migration. Some account is taken of the possible modulation of those health risks by climate change.

Discussion: Climate-change–related migration is likely to result in adverse health outcomes, both for displaced and for host populations, particularly in situations of forced migration. However, where migration and other mobility are used as adaptive strategies, health risks are likely to be minimized, and in some cases there will be health gains.

Conclusions: Purposeful and timely policy interventions can facilitate the mobility of people, enhance well-being, and maximize social and economic development in both places of origin and places of destination. Nevertheless, the anticipated occurrence of substantial relocation of groups and communities will underscore the fundamental seriousness of human-induced climate change.

Climate change is widely projected to cause substantial increases in the scale of human population movement in coming decades. Forecasts of the number of people who will move by around midcentury in response to the effects of climate change vary from tens of millions to 250 million people [Boano et al. 2008; Brown 2007; Christian Aid 2007; United Nations High Commissioner for Refugees (UNHCR) 2009]. The estimate by Myers (2002) that climate change will cause up to an additional 200 million “environ-mental refugees” by 2050 has become a widely accepted figure—although its empirical basis has been questioned (Brown 2008).

Growing concern about climate change as a significant contributor to future population movements arises particularly from awareness of the likelihood of climate-related changes in people’s living and working environments (Tacoli 2009). This knowledge itself affects people’s perceptions of the risks and bene-fits associated with staying versus migrating. Current scientific assessments project that climate change will—to varying extents among different regions and communities—exacerbate morbidity and mortality, reduce incomes, and decrease access to important forms of natural capital. Accordingly, people may choose to move to places perceived as offering a better life.

In this review, we focus on the health dimensions of future climate-related population movements. After a brief discussion of the likely relationship between climate change and population movement, we examine two key themes relating to health, climate change, and population movement: the risks that climate change poses to human health, and how this may contribute to population movement; and the specific health implications of potential climate-related population movements, particularly the health risks to forcibly displaced people, to those involved in resettlement schemes, and to those who migrate to urban areas. Given the nascent status of this research topic, we necessarily draw on previous studies of refugees, people in resettlement schemes, and migrants as analogs for the health issues associated with future climate-related movements. Finally, we discuss the types of research and policy responses needed to address health issues associated with -climate-change–related migration.

## Background: Climate Change and Population Movements

Human migration in response to climatic variation has a long precedence. Climate-driven ecological change greatly influenced the dispersal of early hominids from Africa around 1.9 million years ago, as well as the movement of *Homo sapiens* within Africa around 125,000 years ago and then out of Africa around 80,000 years ago (Finlayson 2005). These migratory flows were shaped by climatic influences on environmental conditions, air temperatures, food and water availability, social structures, and migratory routes (Finlayson 2005; Issar 2010). In recent centuries, there have been many climate-related emigrations, predominantly related to cooling, droughts, and food shortages. Examples extend from recent times, such as the great surge in displacement and migration in early seventeenth-century Europe after the prolonged cold nadir of the Little Ice Age (with its dramatic spikes in food shortages, hunger, epidemics, and wars), the displacements in Europe and the U.S. Northeast during the 1815–1818 climate-and-subsistence crises, and the aftermath of the 1840s Irish famine (Ó Gráda 2009; Oppenheimer 2003; Zhang et al. 2011)—to the much earlier mass migrations in Mesopotamia as drought and famine ensued in the third millennium BCE (before the common era) (Burroughs 2005).

Today, for the first time, human-induced global climate change is beginning to press on populations [Intergovernmental Panel on Climate Change (IPCC) 2007a]. There is now an overwhelming majority consensus among climate scientists that human-generated emissions of greenhouse gases are initiating climatic changes that are unprece-dented in human experience during the Holocene epoch. Human amplification of the natural “greenhouse effect” reflects the scale and intensity of energy use and economic activity that have arisen during the industrial era, along with the accompanying surge in human numbers and consumption levels (particularly in high-income countries), land use patterns, and food production activities over the past century (Lenton et al. 2008; Rockström et al. 2009; Smith et al. 2009). An increasing body of evidence shows that climate change is already affecting natural systems. Of the > 28,000 cases of significant observed changes in terrestrial and biological systems, > 90% had directions of change consistent with impacts expected from global warming (Rosenzweig et al. 2007). On current trajectories, within the next 50 years climate change is likely to reach a critical stage that will be dangerous to the functioning of many aspects of the natural and social environments upon which human societies depend for well-being, health, and survival (Ashfaq et al. 2009; Battisti and Naylor 2009; Heyder et al. 2011).

Climate change poses risks to communities and livelihoods via impacts on ecosystem “goods and services,” loss of arable land, and increased severity and frequency of climate-related disasters (Oliver-Smith 2009). These impacts may cause people to turn increasingly to migration as an adaptive strategy (Black 2001; Castles 2002; Renaud et al. 2007). However, climate change will not act alone in shaping population movement; rather, “it produces environmental effects and exacerbates current vulnerabilities that make it diffi-cult for people to survive where they are” [International Organisation for Migration (IOM) 2009]. For less developed countries in particular, social, demographic, political, and economic stressors—such as high population density, limited economic opportunity, inequi-table distribution of resources and services, poor urban and land use planning, and armed conflict—will coexist with climate risks and influence migration decisions (Tacoli 2009). Hence, disaggregating the impacts of climate change from those of other processes is difficult, particularly slow-onset environmental changes (Adamo 2010).

Research on climate change and movement of people has been dominated by studies seeking to estimate likely population numbers, and the pathways by which movements might occur (e.g., Bates 2002; Lonergan 1998; Myers 2002; Perch-Nielson et al. 2008; Warner et al. 2008). Estimates are based on broad-scale assessments of exposure to risk, rather than systematic evidence about the sensitivity of human movements to particular environmental changes. They have not considered projected demographic and socioeconomic changes over coming decades or the extent to which adaptation may offset climate impacts and hence the need for migration [Asian Development Bank (ADB) 2011; Barnett and Webber 2010; Brown 2008]. The arguments have been largely normative, and the scale of analysis large and hence low in resolution. A frequent assumption is that climate-related population movement, like migration more generally, poses a threat to the integrity of states and their borders (see Tacoli 2009)—and a risk to political stability and hence a source of increased violent conflict [Barnett 2003; Center for Strategic and International Studies 2007; Clark 2007; German Advisory Council on Global Change (WGBU) 2008; Rahman 1999; van Ireland et al. 1996].

Meanwhile, there has been little research on the impacts of climate-change–related migration on “everyday lives,” including health, humanitarian, and equity aspects (Bardsley and Hugo 2010; McMichael et al. 2010). Yet, the health risks posed by climate-related population movements are likely to become a major source of human suffering, disability, and loss of life—an outcome that, currently, appears more likely than the much-debated possibility of increased violent conflict or state failure (Kolmannskog 2008). Consideration of these human-scale outcomes of climate-related movements remains greatly overshadowed by grander geopolitical -narratives (Barnett 2009).

The dynamics of population movement, whether climate related or not, are complex and diverse. The core choices of migration include weighing the risks and benefits of staying versus moving, and the opportunities and constraints relating to the timing and destination of movements (Barnett and Webber 2010; Bates 2002; Renaud et al. 2007). Such movements include internal displacement and international cross-border movement and may be permanent, short-term, seasonal, or circular in nature. The spectrum of migration decisions ranges from forced to voluntary, yet most movements are neither entirely one nor the other. The spectrum in response to climate change is likely to be similar.

Climate change is amplifying the intensity and frequency of extreme weather events such as floods, heat waves, and extreme wind events (Barriopedro et al. 2011; Schiermeier 2011). The relationship between extreme climatic events and migration is complex, but displacement is typically temporary because people tend to return to rebuild housing and livelihoods in places with which they are familiar (Black 2001; Castles 2002; Johnson and Krishnamurthy 2010; Lonergan 1998; Perch-Nielson et al. 2008; Piguet 2008). Such temporary movements are also generally over short distances, within countries, and follow established channels of movement (Bardsley and Hugo 2010; Kolmannskog 2008).

Climate change will also lead to slow-onset changes in climatic and environmental conditions (e.g., sea-level rise, land degradation and loss, declining abundance of fish, contamination of water resources, and degradation of coral) that contribute to loss of important environmental amenity and livelihoods (Adger 2010). Slow-onset environmental changes can be a proximate factor in long-term movement away from a place of origin. For example, in recent decades some pastoralists from the Sahel and Sudan have migrated within national borders in response to drought (Afolayan and Adelekan 1998; Davies 1996; Hammer 2004), and people from southern Tanzania have moved in response to land degradation (Charnley 1997). Recent estimates indicate that sea-level rise of ≥ 1 m could occur by the end of this century, potentially displacing > 20 million people along the coastal regions of Bangladesh, Egypt, and Nigeria (without allowance for adaptive responses or future population growth) (McMichael and Lindgren 2011).

## The Health Impacts of Climate Change: A Factor Contributing to Migration?

Climate change will affect the safety and health of communities and populations globally. Most health impacts will be adverse and will occur via direct exposures (e.g., heat waves, extreme weather events) and less direct influences arising from disruptions to environmental, ecological, and social systems. Indeed, most of the impacts of climate change on physical, ecological, and social systems will affect human health via changes in food yields, freshwater flows and quality, stability of infectious disease patterns, air quality, social cohesion, and family income and livelihoods (Costello et al. 2009; McMichael and Lindgren 2011). The tendency for people to move will be associated particularly with the existence (or perception or expectation) of an increased frequency of serious and extreme weather disasters, food shortages (and associated losses of livelihoods), and water shortage.

Climate change poses risks to food security via reductions in agricultural and fishery yields, especially in already food-insecure regions such as in sub-Saharan Africa and South Asia (Dinar 2007). Changes in flooding and drying cycles, hotter summers, and the spread of drought conditions in some regions are likely to greatly increase risks to agricultural productivity, particularly in lower-latitude countries (Battisti and Naylor 2009; Dai 2010; Easterling et al. 2007). Warming oceans and ocean acidification endanger coral ecosystems and the artisanal, pelagic, and aquaculture fisheries upon which hundreds of millions of people depend for food. Meanwhile, lack of access to safe drinking water is a further major contributor to morbidity and mortality, particularly among children, in developing countries. Changes in rainfall and river flows jeopardize human health via impacts on agriculture, daily hydration, cooking, and domestic hygiene.

Climate change will also influence the geographic range, seasonality, and incidence rate of various infectious diseases, such as malaria, diarrheal diseases, and cholera (Confalonieri et al. 2007). There is some preliminary evidence of climate-related changes in the geographic and seasonal patterns of several infectious diseases over recent decades, including malaria, dengue fever, and tick-borne borreliosis and encephalitis (McMichael and Lindgren 2011; Tanser et al. 2003).

In response to the health and physical safety risks posed by climatic-environmental disasters, both acute and sustained, migration can be a survival strategy. Yet populations in low-income countries whose health is most at risk from climate change (Confalonieri et al. 2007), and where there are often high pre-existing levels of health problems, are used to coping with adverse health outcomes without recourse to migration. It is likely that population movement that is driven substantially by health risks will occur only where those risks are sufficiently serious and widespread. The following example, from the Horn of Africa, is illustrative.

Famines are extreme health crises that cause people to move to avoid hunger and death (Afolayan and Adelekan 1998). The severe drought in 2011 affecting Somalia, Kenya, Ethiopia, and Djibouti has caused tens of thousands of deaths, with high rates of acute malnutrition, particularly among children. Further, the risk of infectious disease (e.g., cholera, measles, malaria, meningitis) has increased because of the combination of malnutrition (weakened immune system), inadequate health care systems, low immunization coverage, lack of clean water, and poor sanitation (Zarocostas 2011). The event may be an extension of a widening regional impact of climate change (Funk et al. 2008). In this region, high rates of migration preexist because of social and political instability and conflict. However, the drought has caused a considerable increase in migration, both within and across international borders. In July 2011, around 1,300 Somalis were reportedly arriving each day at the Dadaab complex in north-eastern Kenya, and nearly 2,000 Somalis were arriving at the Dolo Ado camps in Ethiopia each week (U.S. Agency for International Development 2011). In this instance, migration rates have increased in part because of the substantial health risks associated with famine.

However, migration is not necessarily an indicator of vulnerability: It can be an adaptive response by communities to cope with the effects of climate change (Black 2001; U.K. Government Office for Science 2011). Specifically, the move to a new location can alleviate health deficits from undernutrition or freshwater shortages, avoid the physical dangers of extreme weather events and degraded physical environments, and enhance access to medical facilities (Uscher-Pines 2009). Indeed, movement could be a form of health-seeking behavior writ large.

## Climate Change, Migration, and Health

**Figure 1:**
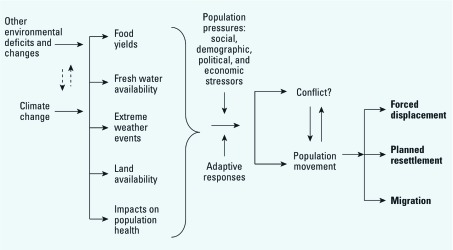
Relationship of climate change to potential population movements. The question mark after “Conflict” refers to the much debated topic regarding whether the effects of climate change, such as changes in food yields or population movement, will increase violent conflict.

Analysis of the complex paths by which climate change influences population movement can be facilitated by using a three-category typology (Figure 1): forced displacement, planned resettlement, and migration.

Forced displacement is likely to occur as environmental changes and extreme climate events undermine peoples’ ability to live in their places of residence. This displacement will typically be over short distances and may involve large-scale movements of people, most often within countries. The first subsection below considers the health implications of displacement, with reference to health outcomes among political and environmental refugees.

There may be planned resettlement of large populations to reduce their exposure to climate impacts. These are likely to be at the scale of communities, resettled within countries. The second subsection focuses on the health implications of planned resettlement schemes, from which the evidence comes largely from large-scale resettlement for dams and environmental remediation.

Migration is also likely in response to, or in anticipation of, climate-related impacts, and will likely be within countries and contribute to urbanization. The third sub-section addresses the health implications of climate--related migration, focusing particularly on those migrating to urban areas within -developing countries.

*Health implications of displacement.* People displaced by climate-related processes and events will include those who have little choice because of loss of habitable land, extreme health risks, and deteriorating live-lihoods (Bardsley and Hugo 2010). This will include people affected by major environmental deterioration and disasters such as floods, landslides, and famine. Where climate change contributes to large-scale displacement, health outcomes can be expected to resemble those of refugees during the early phases of flight and displacement. As with refugees, many of the places that will receive climate-change– affected migrants are in developing regions where public health resources are lacking or inadequate (Carballo et al. 2008).

Forced displacement typically increases the risks of adverse health outcomes, particularly for vulnerable groups such as children, women, the elderly, and those with pre-existing illnesses (Toole 2005). The health risks associated with forced displacement are due to a lack of basic necessities for good health, such as food, shelter, and water, as well as reduced access to health care and loss of social networks and assets.

Among refugees and forcibly displaced people in developing countries, infectious disease is a major cause of morbidity and mortality [International Federation of Red Cross (IFRC) 2007]. After the 2004 Asian tsunami, one million people were initially displaced in Sri Lanka. In the immediate aftermath, there was an increased risk of water and food-borne disease outbreaks (e.g., cholera, dysentery, typhoid fever, and hepatitis A and E), disease related to overcrowding (e.g., measles, meningitis, and acute respiratory infection), and vector-borne disease (e.g., malaria and dengue) [World Health Organization (WHO) 2005]. Infectious disease outbreaks occur particularly in camps or settlements that are crowded, are poorly ventilated, and have inadequate shelter, water, sanitation, and access to immunization and health care facilities (Rajabali et al. 2009). The most common infectious diseases are diarrheal disease, measles, meningitis, acute respiratory infections, tuberculosis, and malaria (De Bruijn 2009; Murray et al. 2009). Malaria-specific mortality rates are especially high when refugees have fled through or into areas of high malaria endemicity, such as highland Rwandans moving to Zaire in 1994 (Toole and Waldman 1997). Where climate-driven disasters contribute to large-scale population displacement, infectious disease is likely to be a significant health problem.

Food shortages, restricted access to food, and undernutrition are recurring problems for displaced people in low-income regions [Toole 2005; UNHCR/World Food Program (WFP) 2006]. The prevalence of acute malnutrition among children < 5 years of age in refugee populations is particularly high (Toole and Waldman 1997). A high incidence of micronutrient deficiency diseases has been reported in refugee camps, including pellagra (niacin deficiency), scurvy (vitamin C deficiency), and anemia (iron deficiency) (UNHCR/WFP 2006). It is likely that much climate-related displacement will occur in food-insecure regions where many people are nutritionally compromised at the outset. Further, climate change itself will negatively affect global food yields, costs, and accessibility as farming, fisheries, and agricultural production are affected by long-term shifts in climatic and environmental conditions and increased adverse weather events (McMichael and Lindgren 2011). An estimate of the burden of disease attributable to climate change at the year 2000 indicated that half of the attributable deaths (predominantly in children) are due to undernutrition (McMichael et al. 2004). Malnutrition, accompanied with low rates of immunization, poor sanitation, and lack of medical facilities in low-income countries, is especially serious given the prevalence of new worldwide pandemics, such as the influenza A (H1N1) virus (Mowafi 2011).

Social instability and displacement present high-risk situations for the spread of sexually transmitted infections, including HIV infection (IFRC 2007). This is attributed to overcrowding, poverty, disruption of family and social structures, increased sexual violence, and limited access to barrier contraceptives and health services and education (Toole 2005). Women in refugee camps face additional reproductive health risks: elevated risks of maternal mortality, unmet needs for family planning, limited access to clinical health services, complications after unsafe abortions, and gender-based violence (Jones 1999; Petchesky 2008; UNHCR 2003).

Research into the mental health of refugees and displaced people has documented elevated rates of mental health problems. Poor mental health is attributable in part to pre-displacement experiences of violence and trauma. However, post-displacement stressors also create substantial mental health risks (McMichael and Manderson 2004; Watters 2001), including fragmented social networks and separation from family, loss of familiar social contexts, poor social connections, diminished sense of belonging, economic deprivation, inadequate housing, little educational and job security, and in some cases mandatory detention (Carballo et al. 2008; Scudder and Colson 1982; Silove et al. 2001; Steel et al. 2004). Further, there are no recognized national or international legal frameworks that address environmental migration (IFRC 2004; Oliver-Smith 2009). Accordingly, people who cross national borders in response to the effects of climate change will have uncertain migration status and will face potentially hostile conditions of reception (Voutira and Dona 2007). This exacerbates risks to mental health.

Finally, although there remains minimal empirical evidence (Black 2001), there is growing speculation about the potential contribution of climate change to violent conflicts (Barnett and Adger 2007; Kolmannskog 2008; McDonald 1999; Reuveny 2007). Should this occur, such conflicts are likely to be complex emergencies that involve poverty, violence, environmental degradation, large-scale health crises, and displacement (Kolmannskog 2008). Population movements may also trigger conflicts and environmental degradation in destination areas, in turn creating further health crises and population displacement (Burkle 1999). For example, climate change has been implicated in violence in southern Sudan: Research suggests that a dramatic fall in average rainfall in south-western Sudan and drought led to increased migration southward, thus contributing to initiation of conflict between nomadic pastoralists and seden-tary farming communities (United Nations Environment Program 2008). Should climate change contribute to conflict, the health impacts will include increased mortality and injuries, gender-based violence, physi-cal and psychological trauma, food shortages and malnutrition, and collapsed primary health services (Mowafi 2011; Toole 2005).

*Health implications of planned resettlement.* Discussions about the risks climate change pose to vulnerable populations—particu-larly those from low-lying islands and coastal deltas—have identified planned resettlement as an adaptation response (Biermann 2010; Byravan and Rajan 2006; Kelman 2008). However, few resettlement schemes have improved the lives and well-being of resettled people (Asthana 1996; Cernea 1997; Kloos 1990). Resettlement schemes for develop-ment initiatives (e.g., dams, hydroelectric schemes) and infrastructure investments in cities (e.g., railway development, building complexes) fail when people have not chosen to be resettled and have no control over the destination and process of movement. There is also concern that powerful actors will use climate change as an excuse to conduct forced migrations for political or economic gain (Barnett and Webber 2010). Accordingly, planned resettlement must be the last resort where other adaptation strategies are ineffective or unavailable (ADB 2011; Barnett and Webber 2010).

Planned resettlement is associated with adverse health outcomes: poor mental health, food insecurity, unsafe water supply, inadequate sewerage systems, and increased infectious disease (Cernea 1999). Resettled populations are exposed to disease vectors for which they have not developed behavioral controls or population-level immunity. For example, people resettled from the highlands of Ethiopia in the mid-1980s were exposed to malaria when they were moved to lowland areas (Kloos 1990). They also experienced higher rates of intestinal parasites, oncho-cerciasis, and yellow fever. Development activities associated with resettlement schemes can also create new vectors for infectious diseases (Kloos 1990). For example, where resettled populations live near newly constructed dams, they become exposed to health risks such as encephalitis, filariasis, gastroenteritis, intestinal parasites, hemorrhagic fever, malaria, and schistosomiasis (Lerer and Scudder 1999).

Resettlement schemes typically lead to adverse social outcomes: landlessness, joblessness, homelessness, social marginalization, heightened food insecurity, loss of access to common property resources, and community disarticulation (Cernea 1997). For example, planned resettlement of low-income households in urban areas can lead to loss of income potential as households are moved to peripheral locations with reduced employment opportunities (Patel et al. 2002). A key outcome of these changes is malnutrition, because the environmental, market, and social conditions under which people previously secured food often differ in new locations (Cernea 1997; Kloos 1990). Resettlement schemes also contribute to social, behavioral, and mental health problems (Good 1996). In indigenous populations in North America and Australia, forced resettlement has created significant cultural disruption, depression, alcoholism, and dietary disorders (Atkinson 1990; Good 1996). These effects have been transmitted across generations. When people who are resettled are disempowered in the process, anger and resentment can arise, which can lead to violence between resettled communities and public authorities and local populations, as well as domestic violence (Good 1996; Thukral 1996). Indeed, even in the case of resettlement of famine-affected communities in Ethiopia in the mid-1980s, “the situation of most settlers was not improved . . . on the contrary, a number of new problems have been caused” (Kloos 1990). For reasons of health alone, planned resettlement to reduce vulnerability should be avoided because such schemes entail substantial risks to health and well-being.

*Health implications of urban migration.* Cities are usually among the most common destinations of migration flows, and environmental changes increase the influx of urban migrants (Adamo 2010). The environmental and economic effects of climate change, such as flooding, water shortages, drought, declines in farm yields, and loss of livelihood, have begun to amplify rural–urban migration in developing regions (Adamo 2010; IPCC 2007b). For example, worsening floods in the Mekong Delta have contributed to increased internal displacement and seasonal mobility to urban centers, notably Phnom Penh and Ho Chi Minh City (Adamo 2010).

Migration provides an adaptive strategy in response to perceived or actual threats associated with climate change (Bardsley and Hugo 2010; Dessai et al. 2004). However, the urban poor, particularly those in crowded settlements with poor water and sanitation facilities, are prone to ill health (ActionAid International 2007; Montgomery et al. 2003). Accelerated population growth, notably the expansion of slums or neighborhoods in vulnerable areas, places stress on urban socioeconomic conditions and facilities (e.g., labor markets, education and health care services, public safety). Poor urban settlements are typically neglected by local or national government authorities and have inadequate infrastructure, high rates of underemployment, and income instability (Campbell 2010).

Further, many urban poor communities are in locations at high risk of climate change impacts: low-lying plains, coastal zones, unstable slopes, and drylands (Campbell 2010; Huq et al. 2007). People migrating to urban poor settlements will face ongoing threats associated with climate change, including water shortages, flooding, sea-level rise, and extreme weather events. For example, in large cities on the east coast of India, morbidity and mortality due to cyclones and storms are expected to rise because of increasing urban poor populations due to rural–urban migration (Revi 2008). Hence, people migrating into these settings may face continued environmental, physical, and psychosocial health threats (Sward 2008).

Although there may be significant risks to health in urban poor areas, health outcomes will be determined in part by who migrates. Demographic studies have noted the “healthy immigrant” effect: those in better health elect to and are positively selected for migration compared with those who are in poor health (Ullman et al. 2011). For example, rural–urban migrants are more likely to be young, employed, better educated, and therefore healthier than non-migrants (Tacoli 2010). Conversely, an unhealthy individual may have less success in crossing heavily militarized borders, such as the Mexico–U.S. border. However, research suggests that the “healthy immigrant” effect is attenuated with increasing duration of residence in host communities.

There is evidence that rural–urban and international migrants are at higher risk of developing chronic diseases, such as cancer, hypertension, coronary heart disease, cardiovascular disease, and type 2 diabetes, compared with those in places of origin (Carballo et al. 2008; Montgomery et al. 2003). For example, among Mexican-born immigrants, length of U.S. residency is directly associated with increasing risk of obesity (Barcenas et al. 2007). The increased incidence of chronic disease after rural–urban and international migration, relative to source populations, has been attributed to changes in diet, acculturative stress, physical inactivity, isolation, and increased health risk behaviors such as smoking and hazardous use of alcohol (Bermingham et al. 1999; Burns 2004; Montgomery et al. 2003; Palinkas 1995; Westermeyer 1993). Poor chronic disease outcomes are also due to lack of access to health care services, including preventative health care and early diagnosis (Uba 1992).

Infectious agents can move with migrants and displaced populations, and this can lead to increased risk of infectious disease (e.g., tuberculosis, hepatitis B, intestinal parasite infections) in host populations (Palinkas et al. 2003). Malaria and dengue pathogens often move with people, and migration from endemic areas can initiate outbreaks or increase transmission in sites of settlement. Similarly, schistosomiasis (for which water snails are the intermediate host organism) is spread by population movement, and this will be of particular concern in areas of increased rainfall and flooding (Carballo et al. 2008). In Australia, the refugee and humanitarian program includes pre-departure screening for infectious diseases (e.g., tuberculosis, HIV) in order to limit exposure to infectious agents among the wider Australian population and, controversially, to limit costs to the Australian health care system. However, in cases of internal and undocu-mented migration, screening for infectious -disease among migrants will be challenging.

Finally, although popular discourse suggests that migration reflects a failure to adapt to climate change, migration is also an adaptive response that will allow migrants, their families, and the communities they move between to cope with the effects of climate change (Agrawal 2008; Bardsley and Hugo 2010; Barnett and Webber 2010). Rural–urban migration is often circular, and migrants continue to maintain links with rural areas and participate in development of regions of origin. Migration can benefit those left behind in environmentally degraded areas, reducing poverty through remittances that can be spent on food, clean water, and health care. Research in Côte d’Ivoire has shown that migrants from Burkina Faso send home remittances that are invested in schools, hospitals, and water and irrigation systems (IOM 2009). Migration can also provide a coping strategy that diversifies and strengthens people’s livelihoods, assets, and incomes, which in turn helps to reduce food insecurity and improves access to health care (Bardsley and Hugo 2010; Black 2001). Although significant challenges are associated with migration, including health risks, migration is also a strategy that can potentially reduce vulnerability.

## Discussion

The primary and appropriate focus of the international policy discourse on human--induced climate change is on the critical issue of reducing carbon emissions through miti-gation efforts. However, progress has been slow, and climate change adaptation has necessarily become an increasingly prominent international policy agenda item (Reid and Huq 2007). Indeed, the need for anticipatory planning for alternative futures in relation to -climate-change–related migration was explicitly recognized in the 2010 Cancun Adaptation Framework [United Nations Framework Convention on Climate Change (UNFCCC) 2010], inviting parties to undertake “measures to enhance understanding, coordination and cooperation with regard to climate change-induced displacement, migration and planned relocation, where appropriate, at national, regional and international levels” (UNFCCC 2010). Migration, which has often been regarded as a major problem by policy makers and governments, is increasingly understood as part of the climate change “adaptation portfolio” (Tacoli and Mabala 2010).

In this article, however, we have argued that climate-change–related migration or involuntary relocation will frequently be associated with poor health outcomes. Health concerns should be central to endeavors to enhance understanding, coordination, and cooperation on population movements due to climate change. Policy making will need to recognize the complexity and heterogeneity of migration—in terms of motivating factors and diversity in duration and destinations and in the demographic and socioeconomic charac-teristics of migrants (Tacoli 2010).

Adaptive strategies to lessen risks, plus public health preparedness, can help build community resilience and reduce vulnerability to climate change. The National Adaptation Programmes of Action (NAPAs) provide an avenue for least developed countries to respond to their climate change adaptation needs (UNFCCC 2012). An analysis of 41 of 49 NAPAs submitted to the United Nations Framework Convention on Climate Change as of May 2009 indicates that many NAPAs explicitly recognize the linkages between climate change and human health, and 18 link climate change to migration (Hardee and Mutunga 2009). However, Agrawal’s (2008) review of the NAPAs found only two examples in which mobility was identified as an adaptation strategy.

Faced with competing priorities and climate change challenges, the NAPA process compels countries to prioritize projects geared to immediate priorities in single sectors (e.g., alleviation of water resource scarcity) (Hardee and Mutunga 2009). Given the complex paths by which climate change will affect human health and migration responses, effective public health and adaptation strategies must embrace policies across multiple sectors, including health, water, agriculture, energy, and transport. This will require coordinated efforts of local and national governments and relevant international, national, and local institutions, non-government organizations, and agencies. Adaptive strategies, including public health initiatives, should also be integrated with existing national development, public health, and poverty reduction strategies. Clearly, there is a need for multilevel, interdisciplinary, and integrated adaptation measures and emergency responses—and for funding bodies to recognize the eclectic nature of this need.

Public health and policy responses to address health risks associated with climate-related migration must be responsive to the nature of mobility and the demographic charac-teristics of those who move. Broadly, it is important to *a*) minimize any health inequities and ensure access to services, *b*) ensure health rights of migrants, *c*) implement interventions to reduce excess mortality and morbidity among migrant populations, and *d*) minimize the negative impact of the migration process on migrants’ health outcomes (WHO 2010). Meanwhile, much can be learned from the existing evidence base and policy and programmatic responses in the area of large-scale population displacement (e.g., after humanitarian crisis and disaster), planned resettlement schemes, and urbanization (specifically settlement in urban poor areas in developing countries).

There is an extensive and well-established international regime for responding to large-scale humanitarian and environmental disasters, such as conflict, droughts, and floods. A fundamental role of public health and disaster relief services—provided by government authorities, international humanitarian agencies, UN organizations, and local and international non-government organizations—is to assess health needs, allocate resources, and provide health services. Of fundamental importance are the accessibility and quality of health care, health education, and prevention measures; establishment of adequate disease surveillance systems; control of pathogenic agents; targeting “hidden” populations; monitoring and evaluating health interventions such as vaccination coverage; participation of affected populations in health care planning; coordination between services and administrative bodies and target populations; and consideration of the health needs of host populations (Mowafi 2011).

These challenges will remain central to public health responses in the case of climate-change–related population displacement, particularly during sudden-onset disasters. Pre-disaster planning can increase the impact of international assistance and aid and decrease the public health tolls (Benjamin et al. 2011). To respond to potential increase in large-scale disasters, it will be necessary to build on existing frameworks that seek to prepare for, respond to, and recover from public health issues in situations of large-scale population displacement (e.g., Inter-Agency Working Group on Reproductive Health in Crises 2010; Sphere Humanitarian Charter and Minimum Standards in Disaster Response 1998).

Planned resettlement to reduce climate change vulnerability will often involve substantial risks to health and well-being. Where necessary, the social and health costs of resettle-ment can be minimized by allowing adequate time for community consultation and planning, paying compensation at a level equal to the standard of housing and materials in the host community, ensuring that the money and resources made available to assist communities to relocate is spent on those communities, avoiding payments to inter-mediaries, employing the people being moved wherever labor is required, and providing support for housing, health services, mental health services, employment, and education. As far as is possible, populations must want to move and must have active influence in all stages of the decision-making and resettlement processes (Barnett and Webber 2010; Biermann and Boas 2008; Cernea 1997; Cernea and McDowell 2000; Johnson and Krishnamurthy 2010). The bene-fits of such conditions are clearly illustrated in the resettlement of 60,000 low-income people living on illegally occupied land adjacent to the railway tracks in Mumbai during the late 1990s and early 2000s to allow construction of an improved rail service. Although not without some problems, this resettlement scheme did not impoverish those who moved; the actual move was voluntary; resettled people now live in secure, better-quality accommodations with provision for piped water, sanitation, and electricity; and, importantly, the resettled people were involved in designing, planning, and implementing the program and managing the settlements to which they moved (Patel et al. 2002).

Migration to poor urban and peri-urban areas, often to informal settlements, is anticipated as a frequent outcome of climate change. Risks to health will result. There are well-documented inequities in health care and status between vulnerable within-country migrant groups and host populations in developed countries [United Nations Development Program (UNDP) 2009]. Urban policy makers and services in low- and middle-income countries should therefore focus on improving environmental conditions and access to health services to improve health outcomes, including emerging health issues such as chronic noncommunicable diseases, among the urban poor (Montgomery et al. 2003). Specifically, urban planning and public health should promote access to healthy foods (including local low-impact food production), good air quality, conservation and decontamination of land, protection from excessive noise, good water and sanitation, physical activity, social cohesion, housing quality, access to employment facilities, community and road safety, and the reduction of poverty (Global Research Network on Urban Health Equity 2010). There has also been a shift toward integrated and participatory approaches to urban development and planning. For example, the Slum Networking project in Ahmedabad, India, has addressed physical, social, and economic development needs utilizing decentralized governance (i.e., participation by stakeholders from the public, private, and nongovernmental sectors) and community participation models (Das and Takahashi 2009).

The health needs and outcomes of -climate-related displaced populations will depend on the nature of migration processes and the capacity of receiving communities and countries. There are many innovative policies and interventions that address health issues and access to health services for international immigrants, including migrant-focused health schemes, the European “Migrant-Friendly Hospitals” project, and refugee and migrant-focused health services (UNDP 2009; Wirtenberger et al. 2004). International coordination mechanisms have been developed to address migration-related health issues, including the United States–Mexico Border Health Commission and, in Europe, the Northern Dimension Partnership in Public Health and Social Well-Being. However, international migration accounts for only a small proportion of all current population movement, and it is likely that most climate-change–related population mobility will be internal, and mostly in developing regions that lack the resources to respond fully to the health needs of displaced people (Mowafi 2011). The inclusion of migrants and displaced people in health systems will therefore require multisectoral and effective coordination between non-governmental organizations, United Nations agencies, and governments. A key component, particularly in lower-income countries, will be to support public health capacity and action, health surveillance, and data collection.

Finally, there is a heightened need for research and data collection systems that can capture information relating to population movements and migrant health indicators and needs. Data collection and research will be demanding, because displaced popu-lations can be difficult to reach as people move both within and across multiple geographic borders and through formal and informal “bureaucratic” regimes. Key roles for data collection systems and research are to identify ways of reducing vulnerability in climate-change–affected communities in order to reduce the likelihood of displacement and maintain good health, understand the risks to health during migration and resettlement processes among climate-affected populations, and identify and evaluate interventions and policies that will improve health outcomes among those who move. Such research can inform policy interventions, research, and public health practice with the goal of increasing the health and health equity among -climate-change–related migrant populations.

## Conclusion

If climate change continues on its current trajectory, then an increase in the numbers of displaced people over the coming decades is likely. Although the range and extent of health risks associated with future climate-related population movements cannot be clearly foreseen, the evidence of health outcomes of analogous movements of people indicates that health risks will predominate over health benefits. This, then, is an issue of considerable geopolitical, ethical, and economic importance.

Planned resettlement and migration will usually be last resort options. However, effective policy must facilitate the mobility of people when it becomes necessary, thereby seeking to enhance well-being and maximize social and economic development in both the places of origin and destination (Bardsley and Hugo 2010). Whether migration can become an adaptive strategy or a last resort option depends on proactive policy decisions today (ADB 2011). Beyond self-interest and ethical concerns is the moral challenge inherent in the fact that, historically, today’s wealthy industrialized countries have caused most of the current excess accumulation of atmospheric greenhouse gases and should therefore take substantial responsibility for assisting climate-related migration (Biermann and Boas 2008).

Climate change mitigation, through reduction of greenhouse gas concentrations, is the primary aim for governments and communities. A fuller understanding of the range of risks posed by climate change to human well-being and health, including the health of those involved in climate-related migration, could strengthen the resolve of the international community to take effective mitigation action to abate climate change. Yet, because climate change is already occurring and at least some future temperature changes are already “locked in,” adaptive strategies are also required to protect against increasing risks to human health and the adverse effects of climate-related migration and displacement. Through deep cuts in global greenhouse gas emissions, together with comprehensive socioecological adaptation strategies, adverse climate-related migration and health outcomes could be greatly reduced.
